# A bionic bird jumping grasping structure design based on stm32 development board control

**DOI:** 10.1038/s41598-024-61285-y

**Published:** 2024-05-07

**Authors:** Chunpeng Zhang, Weiping Shao, Yongping Hao

**Affiliations:** 1 School of Mechanical Engineering, Shenyang Ligong University, Shenyang, 110159 China; 2 School of Equipment Engineering, Shenyang Ligong University, Shenyang, 110159 China; 3 Liaoning Key Laboratory of Advanced Manufacturing Technology and Equipment, Shenyang, 110159 China

**Keywords:** The bionic bird, Lower limb structure, Bouncing grab, stm32, Engineering, Mechanical engineering

## Abstract

During takeoff and landing, birds bounce and grab with their legs and feet. In this paper,the lower limb structure of the bionic bird is designed with reference to the function of jumping and grasping, and the PID algorithm based on the development module of stm32 development board is used to speed control the lower limb driving element, so that the motor and the bishaft steering gear move with the rate change of sine wave. According to the speed of grasping response time and the size of grasping force, the structure of the bionic bird paw is designed. Based on the photosensitive sensor fixed in the geometric center of the foot, the grasping action of the lower limb mechanism is intelligently controlled. Finally, the kinematic verification of the lower limb structure is carried out by ADAMS. Experiments show that the foot structure with four toes and three toes is more conducive to maintaining the stability of the body while realizing the fast grasping function. In addition, it can effectively improve the push-lift ratio of the bionic ornithopter by adjusting the sinusoidal waveform rate of the motor speed.

## Introduction

With the development of bionic birds in the field of aviation, research on high bionic performance of bionic birds has increasingly become the mainstream of related scientific research fields. Most studies on the legs and feet of birds in this field are based on the bionic design of legs on the UAV equipped with rotorwing, and the bionic performance of such designs is not perfect and the concealability of the body is not high. Therefore, the assembly form of bionic lower limbs loaded on the bionic bird body will become a future development trend^1^.

Based on the analysis of the movement posture characteristics of the take-off legs and feet of birds, it can be seen that the tibiotarsal bones and the metatarsal bones perform relative rotation like gear meshing when the legs of birds are curled up for energy storage^2^. The feet grasp the roosting branches. When the body reaches a certain Angle with the ground, the body posture is adjusted to a certain inclined upward Angle with the ground, and the jump is instantaneous^3,4^. At the same time of the jump, the grasping posture of the feet is lifted, and the wings are spread to flutter, and the jumping take-off is finally realized^5^. In the process of landing and roosting, the bird body is in a gliding position and reduces its speed to a controllable threshold range. It reaches forward with its feet, leans back with its body and flaps its wings, and finally reaches the designated roosting place for landing buffer and grab^6^, thus completing the landing and roosting process.

For the design of the functional structure of the legs, the Swiss Federal Institute of Technology designed a contact leg grasping structure combined with the landing and roosting function of birds, and carried out several groups of landing grasping experiments, with a success rate of 80%^7^. However, the grasping structure of the foot is very different from the actual structure of the bird's claw, and the leg structure and the frame cannot be bent and stretched, so it can be seen that its application is mostly suitable for the situation of no wind and low speed. A few years ago, in the research on the legs and feet of bionic birds, Olin College of Engineering for the first time combined the structure of legs and feet with the four-rotor UAV. The legs can be telescoped and folded, and the feet can be grasped, thus realizing the basic simulation performance of the take-off and landing of the bird body to a certain extent^8^. The slight deficiency is that the foot structure is not simulated enough, and the flight carrier is not consistent with the actual flight situation of birds. Later, the foot structure was improved by Stanford University, making the foot structure more realistic to the actual foot claw structure, but the disadvantage is also the similarities and differences of flight carriers^9^. In order to realize the folding and jumping action of the lower limb and leg structure, the domestic bionic locust adopts the coordination of non-full-tooth gear and spring rod structure^10–12^. This kind of direct drive transmission form of motor gear requires high strength of parts, and the life of connecting parts is relatively short. Based on this, this paper designs a lower limb leg and foot mechanism with foldable legs and grasping claws, which is closer to the actual structure of birds and is equipped on the body of a space-fixed wing flapping wing bird.

## Structures of legs and feet of lower limbs

**Figure 1:**
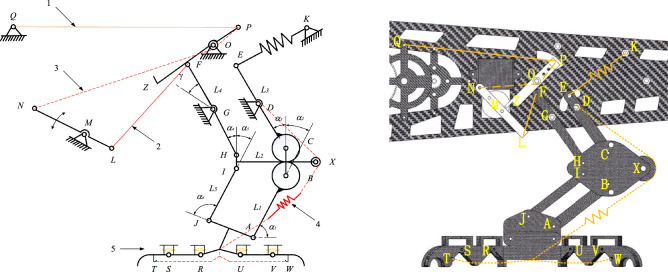
Ultimate pose of leg structure before jumping.

**Figure 2:**
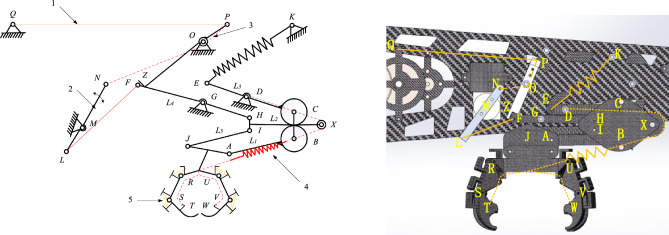
Ultimate position position of jumping preparation.

The function of the lower limb structure is ejection and grab. The ultimate position of the leg structure before takeoff is shown in Fig. 1 and the ultimate position of the bouncing preparatory action is shown in Fig. 2.

Figure 1 is the ultimate pose diagram of the leg structure before the jump of the one-legged four-toed two-toed joint. The leg structure of the lower limb is based on the space parallelogram mechanism and driven by the space rod system and the rope to simulate the leg expansion and contraction action. The feet are passively driven by the space pulley, spring and rope to grasp the space object.

In Fig. 1, the relatively fixed fulcrum *G, D, M, O, P, Q,* and *Y* are firmly connected to the fuselage of the bird body, and each fulcrum floats randomly. When the bionic bird rears its legs in the air after jumping into the air and flying smoothly, the fuselage structure is fixed relative to the legs and feet, and the relative relationship between the fuselage and the legs and feet is the same during the process of gliding and landing. In the process of bouncing takeoff and landing buffering, the feet are fixed relative to the legs and fuselage because of the space fixed grasp of the feet. In Fig. 1, the main drive is driven by a simplified rod *LN* of the steering gear rotating around the relative fixed fulcrum *M*. When in the position shown in the figure, 2 between the rotating pairs* L* and *F* is a tight rope, and the dotted line 3 connecting the rotating pairs *N* and *P* through the fixed pulley *O* is also a tight rope, and the orange solid line 1 between the fixed fulcrum *Q* and the rotating pair* P* is a stretched rubber band. The spring composite dotted line 4 which is fixedly connected to the fixed fulcrum *D* and connected to the foot claw *T* and *W* around the space fixed pulley *X* is a spring rope structure. The member *CE* and member *AB* are non-full-tooth gear rods, and the inner and outer leg structures are connected by the leg floating frame *BCHI*. The part that constitutes the sole of the foot is derived from the rod *AJ* to the I-shaped rod that connects the joints *R* and *U*, while the toe part is an open-ring rod system structure that is connected step by step by step by the rotating pairs *R*, *S*,* U* and *W*. The member 5 shown in Fig. 1 is a rubber band. In order to prevent the toe part from producing joint inversion, a T-shaped solid joint structure is designed at the upper part of each toe segment, and elastic locking is performed by the rubber band. When the spring rope composite structure 4 is stretched, the rubber band is also stretched to store elastic potential energy, preparing for the rapid release of the bird's paw in the future.

When the body performs the bounce action, the steering gear rod *LN* rotates clockwise around the fixed fulcrum *M*, and the taut rope *FL* traction rod *FH* rotates counterclockwise around the fixed fulcrum G. Due to the spatial parallelogram characteristics of the leg structure, the gear rod *CE* is driven to rotate synchronously counterclockwise around the fixed fulcrum *D*, and the spring at the rotation pair *E* and the fixed fulcrum *K* is deformed and stretched to store energy. Provides bounce power for subsequent bounce takeoff. The gear rod *AB* engaged by 1:1 transmission ratio gear engages in clockwise constant speed meshing transmission, which is transmitted to the rod *AJ*, and then drives the rod *IJ* to rotate synchronously with the gear rod *AB* around the fulcrum *I*. In the spring rope composite structure 4, the mechanism motion drives the originally tautted system 4 to stretch, and the spring is stretched to a certain shape variable and then passively forces the lower side rope to tighten, driving the foot claw to grab.

In order to avoid the work of the steering lever *LN* over the *EK* spring, and to ensure the service life of the rope, a mechanism locking system is introduced in this mechanism.The movement changes of the mechanism locking system before and after the execution function are shown in Figs. 1 and 2.

The system is composed of rubber band, rope and rod *PZ*. In the above bouncing process, due to the clockwise rotation of the steering gear rod *LN*, the rope *PN* is relaxed and the rubber band *PQ* rebounds. The lever principle drives the rod *PZ* to rotate counterclockwise around the fixed fulcrum *Y* until the rod *PZ* contacts with the rotating pair *F*. At this moment, due to the flexibility of rubber band 1, the rotation of the rod *FH* will drive the rod *PZ* to rotate clockwise in contact with the rotating pair *F* at all times, until the rotating pair *F* is in vertical contact with the structure at *Z* of the rod *PZ*, so that the reverse force transmitted by the rotating pair is applied to the fixed fulcrum *Y* along the direction of the rod *ZP*, and the structure is finally locked. At this moment, the motor can reverse a small Angle to make the rope *FL* slack, the steering gear can no longer bear too much torque, and the life of the rope *FL* can be guaranteed to a certain extent. The above is the bouncing flexion leg movement process of the lower limb structure.

During the burst spring action, as shown in Fig. 2, the driving steering gear rod *LN* rotates counterclockwise around the fixed fulcrum* M*, and the rope *NP* is pulled to tighten the driving rod *PZ* for clockwise rotation, so as to overcome the friction force between the rotating pair *F* and the end *Z* of the rod *PZ* and do work when the two are separated, leaving enough movement space for the rod *FH* and avoiding interference between the two. Due to the counterclockwise rotation of the driving gear lever *LN*, the rope *LF* is relaxed, and the relaxation length is just enough to meet the tension length of the rotation pairs F and *L* after the spring. Spring *EK* releases the stored energy instantly when the locking mechanism is released, making the leg parallelogram mechanism develop. The spring of the rope spring compound mechanism 4 releases the stored energy, providing additional leg jumping force for the bird body. After the spring recovers its original length, the paw is driven to expand due to the elastic force of the rubber band 5. At this moment, the tension of the spring is lower than the system rebound of the rubber band group, and then the whole bouncing grasp process is completed.

## Design of the number of foot knuckles

**Figure 3:**
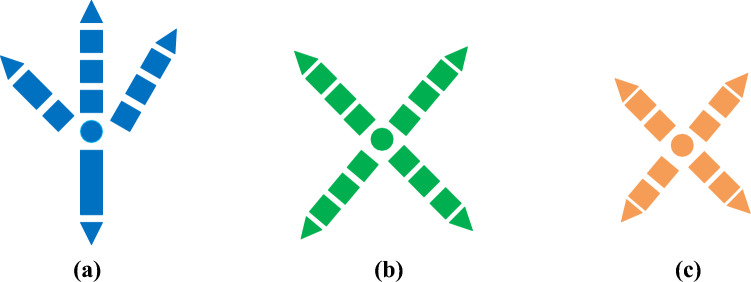
Common bird claw claw type.

In nature, the most common bird feet are four-toed with two-knuckle structure,four-toed with three-knuckle structure, and four-toed compound multi-knuckle structure^13^. In order to achieve the purpose of maintaining all-round stability and stable grasp of the body, the characteristics of the geometric distribution characteristics and functions are evaluated, and the structure of the four-toe three-toe joint is better, and the claw type is shown in Fig. 3.

**Figure 4:**
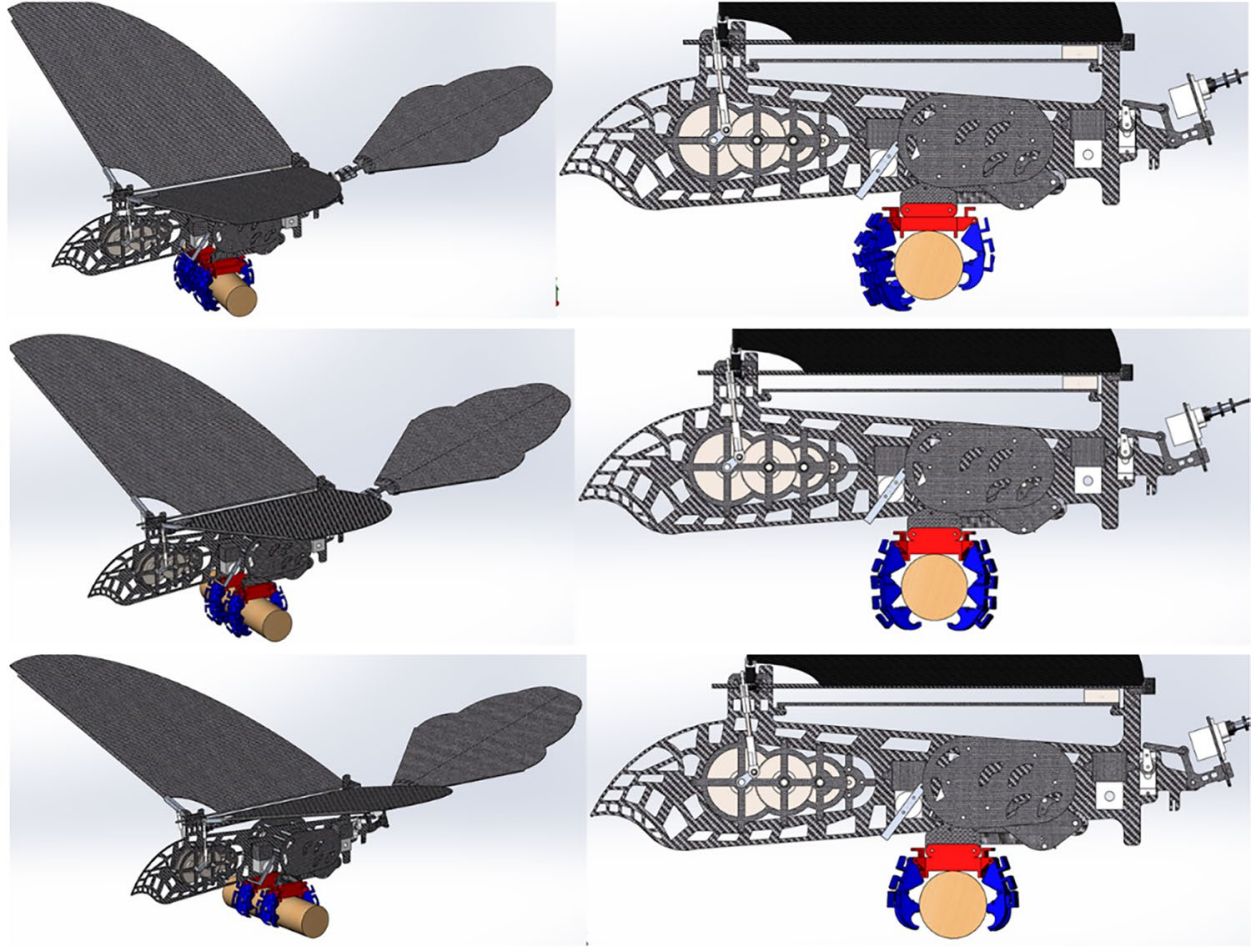
Three types of claw 3D models.

Figure 3a shows the four-toed compound polydactyly structure, mostly of the peregrine claw type. Figure 3b shows the structure of four toes and three toes, mostly woodpecker claw type. Figure 4c shows the structure of four toes and two toes, mostly of parrot claw type.In order to verify the grasping performance of the claw type, this paper carries out model reproduction for the claw type structure, and its three-dimensional model is shown in Fig. 4.

**Figure 5:**
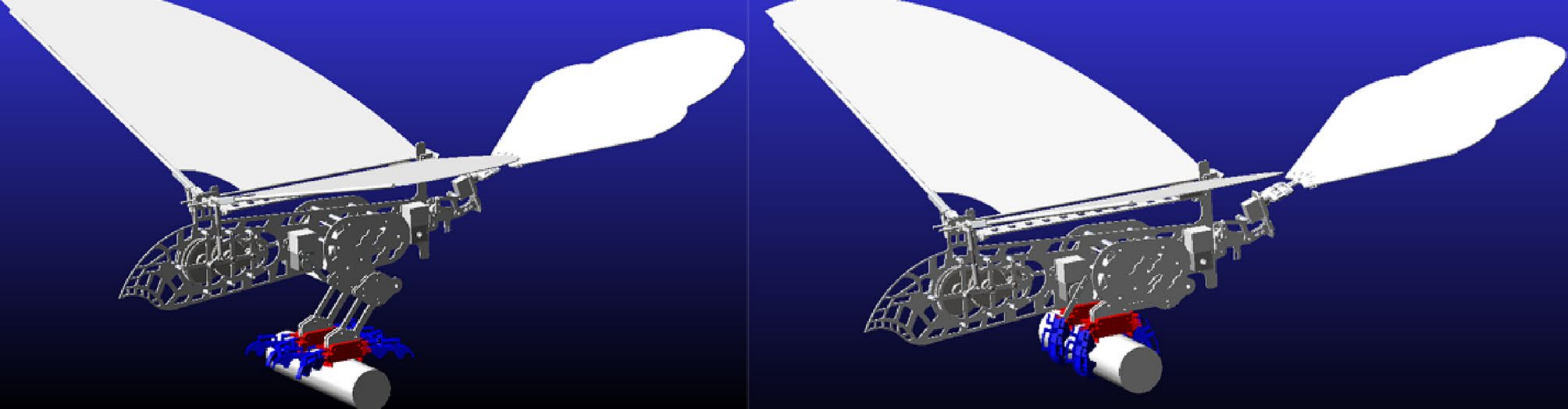
Flapping wing grab motion in software Adams.

**Figure 6:**
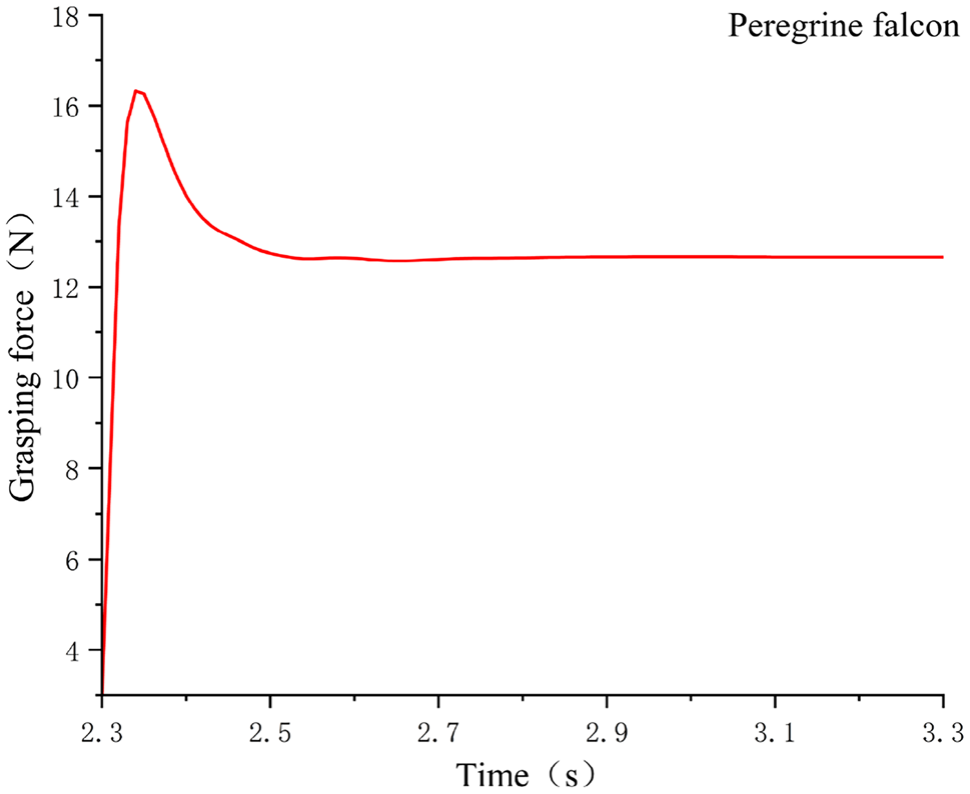
The grasping force of peregrine—shaped claws.

**Figure 7:**
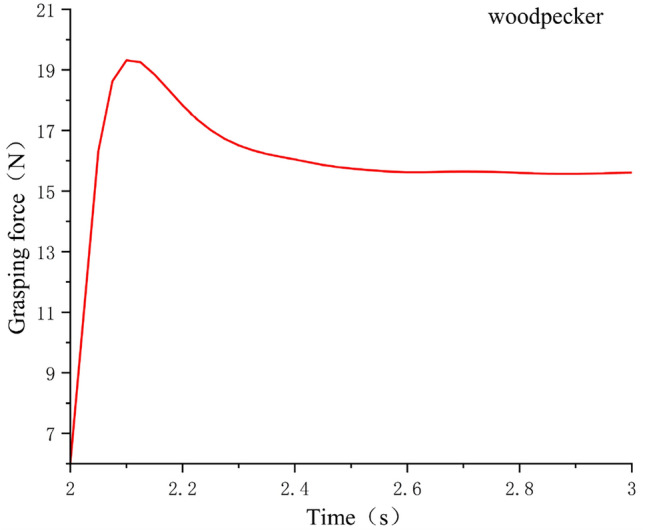
The grasping force of woodpecker—shaped claws.

**Figure 8:**
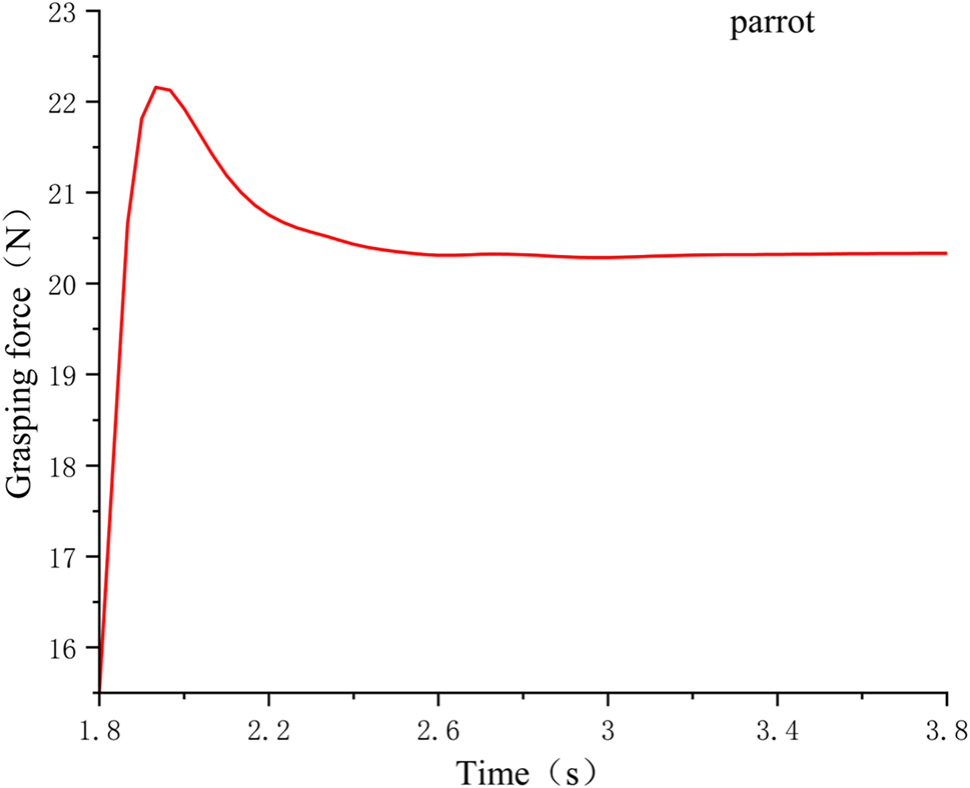
The grasping force of parrot- shaped claws.

In order to verify the rationality of the woodpecker claw structure, the grasping simulation experiments of the same object were conducted for different claw types,The model was imported into the simulation software Adams, and the grasping process between paw and log was analyzed, including the grasping time, grasping state and instantaneous contact force measurement at the contact point. The grasping posture of the bionic flapping wing in the software is shown in Fig. 5, the grasping curves of the three claw types are shown in Figs. 6, 7 and 8, and the experimental data were shown in Table 1.

**Table 1:** Capture experimental data.

Claw type	Grab objects	Grasping effect	Grasping time/S	Grasping force/N
Peregrine falcon	5 cm diameter cylindrical branches	All wrapped and grabable	2.3	12.9
woodpecker	5 cm diameter cylindrical branches	All wrapped and grabable	2	15.7
parrot	5 cm diameter cylindrical branches	Half wrapped and ungrabable	1.8	20.4

It can be seen from the experimental data in Table 1 that the parrot claw type is not suitable for grasping objects beyond its travel range. Although it has the largest grasping force, the fuselage cannot maintain stability after grasping, so it is not suitable for the mechanical bird claw type design required in this paper. Compared with the grasping time and grasping strength of Peregrine falcon claw type, woodpecker claw type is better and more in line with expectations, so the design of the claw type in this paper adopts woodpecker claw type.

## Simulation of foot grasping structure

**Figure 9:**
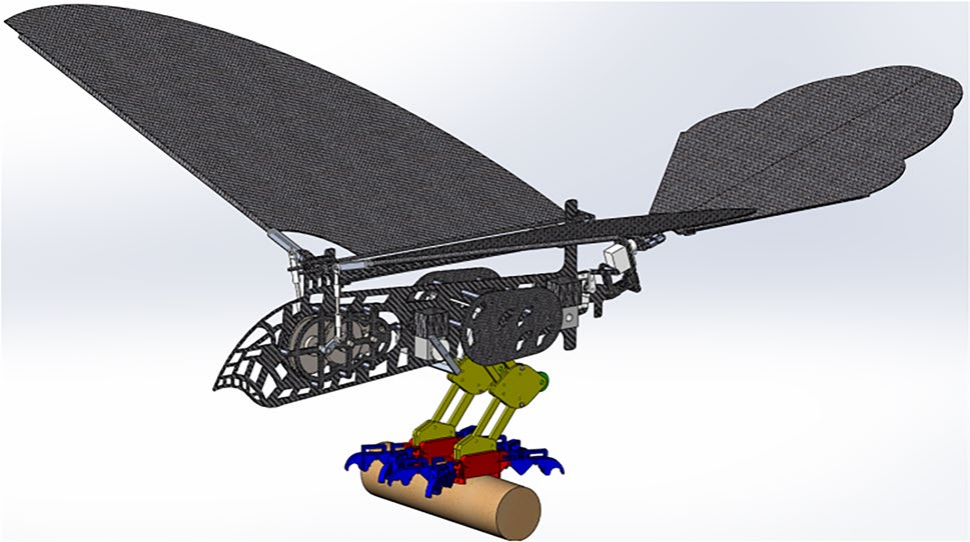
Overall structure of the prototype.

**Figure 10:**
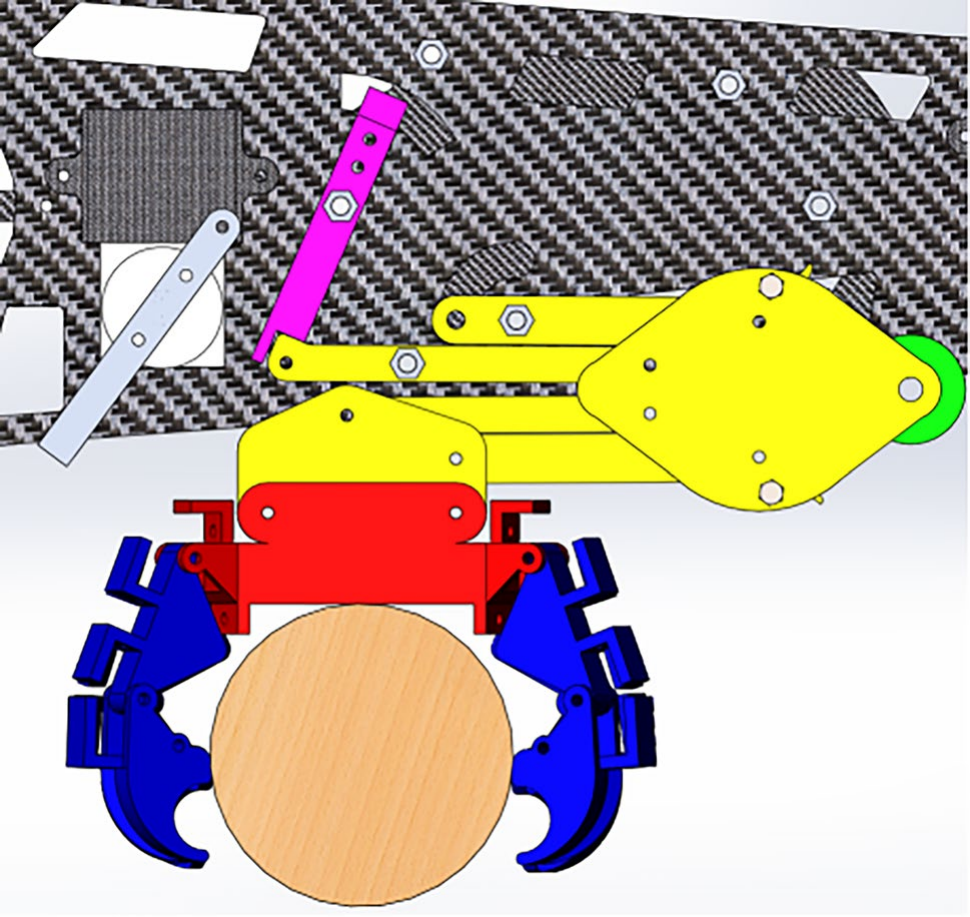
Transmission close-up of lower limb structure.

In order to verify the rationality of the motion of the lower limb grasping mechanism, virtual simulation based on ADAMS was carried out. The overall structure of the prototype is shown in Fig. 9, and the transmission close-up of the lower limb structure is shown in Fig. 10.

**Figure 11:**
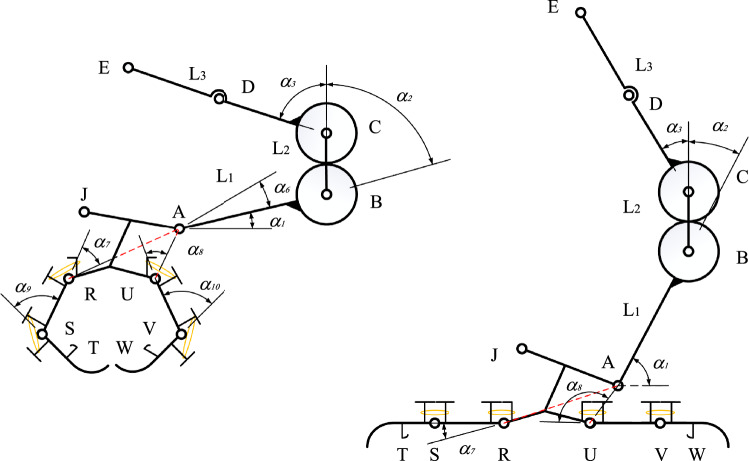
Structure diagram of bar system.

The response speed of the grasping process depends on the angular velocity of FH and the response angular velocity of the end joint of the paw. In order to capture the angular velocity required by the paw joint, D-H coordinate system was established for the legs and feet. Due to the synchronization of the gear rod CE and the gear rod FH as well as the symmetry of the structure, the linkage structure of the outer gear meshing rod was extracted from the legs for analysis, and the linkage structure was shown in Fig. 11.

In Fig. 14, in order to simplify the ankle joint structure, a red dotted rod AR was used to replace the I-shaped rod AJRU. The transformation model from joint S to joint E is as follows:

1 $$ {}_{S}^{E} {\mathbf{T}} = {}_{S}^{R} {\mathbf{T}} \cdot {}_{R}^{A} {\mathbf{T}} \cdot {}_{A}^{B} {\mathbf{T}} \cdot {}_{B}^{C} {\mathbf{T}} \cdot {}_{C}^{E} {\mathbf{T}} $$

$$ {}_{S}^{E} {\mathbf{R}} = \left( {\begin{array}{*{20}c} {c\theta_{1} } & { - s\theta_{1} } & 0 \\ {s\theta_{1} } & {c\theta_{1} } & 0 \\ 0 & 0 & 1 \\ \end{array} } \right) $$

$$ {}_{S}^{E} {\mathbf{P}} = \left( {\begin{array}{*{20}c} {L_{TS} \cdot c\theta_{1} + L_{SR} \cdot c\theta_{2} + L_{RA} \cdot c\theta_{3} + L_{1} \cdot c\theta_{4} + L_{2} \cdot c\theta_{5} + L_{3} \cdot c\theta_{6} } \\ {L_{TS} \cdot s\theta_{1} + L_{SR} \cdot s\theta_{2} + L_{RA} \cdot s\theta_{3} + L_{1} \cdot s\theta_{4} + L_{2} \cdot s\theta_{5} + L_{3} \cdot s\theta_{6} } \\ 0 \\ \end{array} } \right) $$

$$ {}_{S}^{E} {\mathbf{T}} = \left( {\begin{array}{*{20}c} {{}_{S}^{E} {\mathbf{R}}} & {{}_{S}^{E} {\mathbf{P}}} \\ {\begin{array}{*{20}c} 0 & 0 & 1 \\ \end{array} } & 1 \\ \end{array} } \right) $$

$$\theta_{1} = \alpha_{2} + \alpha_{3} + \alpha_{6} + \alpha_{7} + \alpha_{9}$$, $$\theta_{2} = \alpha_{2} + \alpha_{3} + \alpha_{6} + \alpha_{7}$$, $$\theta_{3} = \alpha_{2} + \alpha_{3} + \alpha_{6}$$, $$\theta_{4} = \alpha_{2} + \alpha_{3}$$, $$\theta_{5} = \alpha_{3}$$, $$\theta_{6} = 0$$, $$c\theta_{i} = \cos \theta_{i}$$, $$s\theta_{i} = \sin \theta_{i}$$, $$i = 1\sim 6(i \in Z)$$.

The above is a general matrix formula for calculating the data of the ultimate pose change of the lower extremity structure, in order to more clearly express the specific movement of the lower extremity bar system structure. The detailed parameters are shown in Table 2.

**Table 2:** Kinematic parameters of linkage structure.

	Joint	Length of link/(mm)	Joint angle/(°)	Range of joint angle variation/(°)
Kinematic chain	S	L_ST_	α_9_	0 ~ 70.8
	R	L_SR_	α_7_	0 ~ 42.3
	A	L_RA_	α_6_	18.2 ~ 18.2
	B	L_1_	α_2_	29 ~ 90
	C	L_2_	α_3_	29 ~ 90
	E	L_3_	0	0

According to the boundary conditions of the limit position of the lower extremity structure of the bird, the relative position change relationship between the joints can be obtained by bringing the parameter values into formula (1). The measurement of the required observed angular velocity is based on the kinematic solution of the above motion model.

The initial condition is the response speed of driving the steering gear. In this experiment, the leg rewinding time is 2S, and the speed regulation is approximately triangular waveform. Under the resistance of a single rubber band at each counter-joint, the simulation data shows that the fastest response time at the end of the paw is 1.2S, and the maximum relative angular velocity is 0.3 r/s.

**Figure 12:**
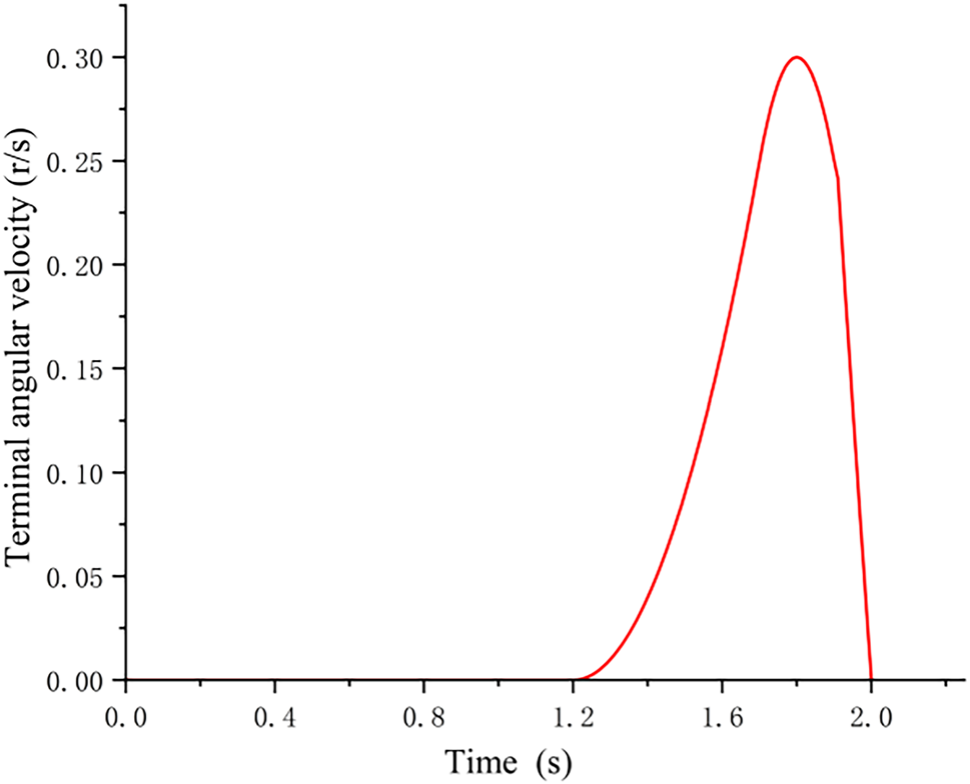
Change curve of the angular velocity at the end of the paw.

The change curve of the angular velocity at the end of the paw is shown in Fig. 12.

As shown in Fig. 12, the paw at 1.2S responds quickly, and the angular acceleration increases continuously. After 1.7S, the rubber band at the antijoint begins to stretch, the angular acceleration begins to decrease, and the paw continues to accelerate. At 1.8S, the paw angular speed reaches the maximum, and then the mechanism decelerates. At 1.9S, the leg bar enters the geometric concave of the locking device, causing the foot angular speed to shake and turn, and the paw angular speed is rapidly decelerated, and finally the mechanism is locked by the locking device, and the speed is reduced to 0 when 2S, and then the grasping action is completed. For the fast bouncing function, it is under the joint action of the steering gear and the locking device to complete the specified action and achieve the expected function, and the design idea of the locust imitation robot is also the same. The speed of the bounce response is related to the rope NP and the spring EK, the length of the rope NP should be able to ensure that there is enough rope tension margin when the burst rebound, and the stiffness of the spring EK should ensure that the body can store enough elastic potential energy under the premise of not breaking the rope, and finally make the body reach the predetermined bounce height. For the actual bones of birds, the curved structure of the bones will reduce the strength of the bones and improve the predictability of the load^14^. The curved bones can offset the impact of external loads, so that the stress value transmitted to the bones is low^15–18^. Based on this, the transmission mode of lower limb structure is designed.

**Figure 13:**
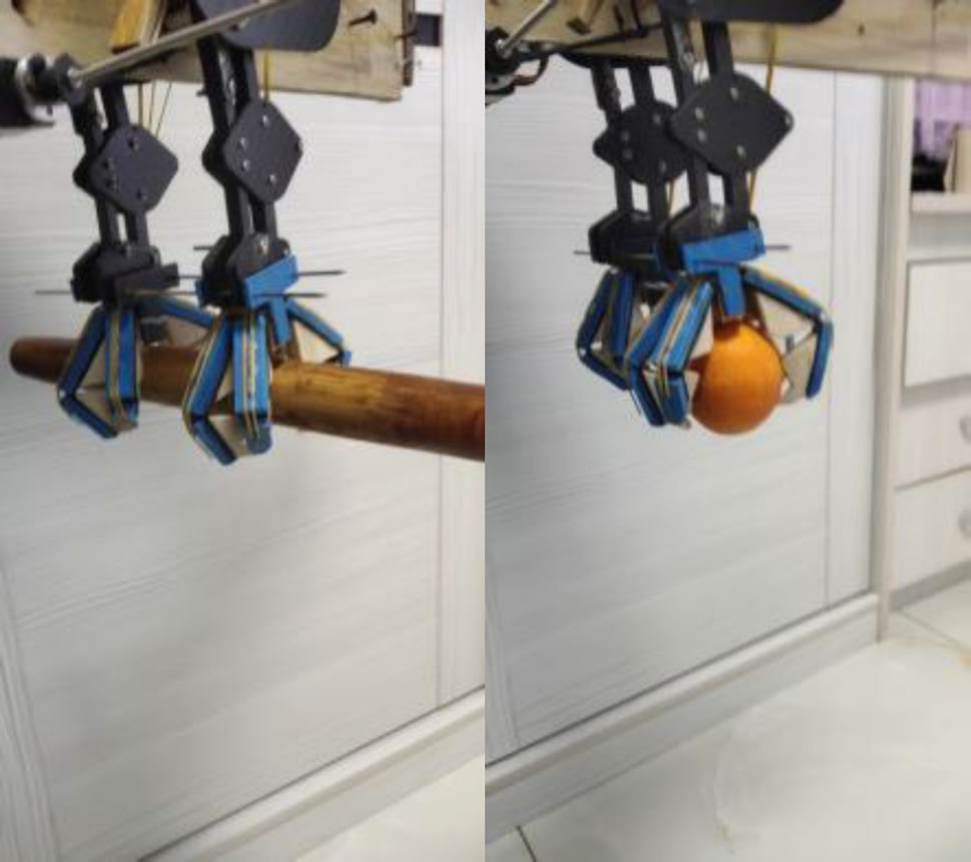
The grasping effect of the claw.

In order to verify the rationality of the design of the lower limb mechanism of the bionic flapping wing, the real limb mechanism was equipped and the grasping test was carried out. The main grasping objects of this test were round sticks with a diameter of 5 cm and an orange. The grasping effect of the claw is shown in the Fig. 13.

Through the test experiment and the actual grasping effect, we can see that this set of lower limb grasping structure can realize the grasping function of imitation birds, and can grasp various forms of objects.

## Body control module and flutter experiment

**Figure 14:**
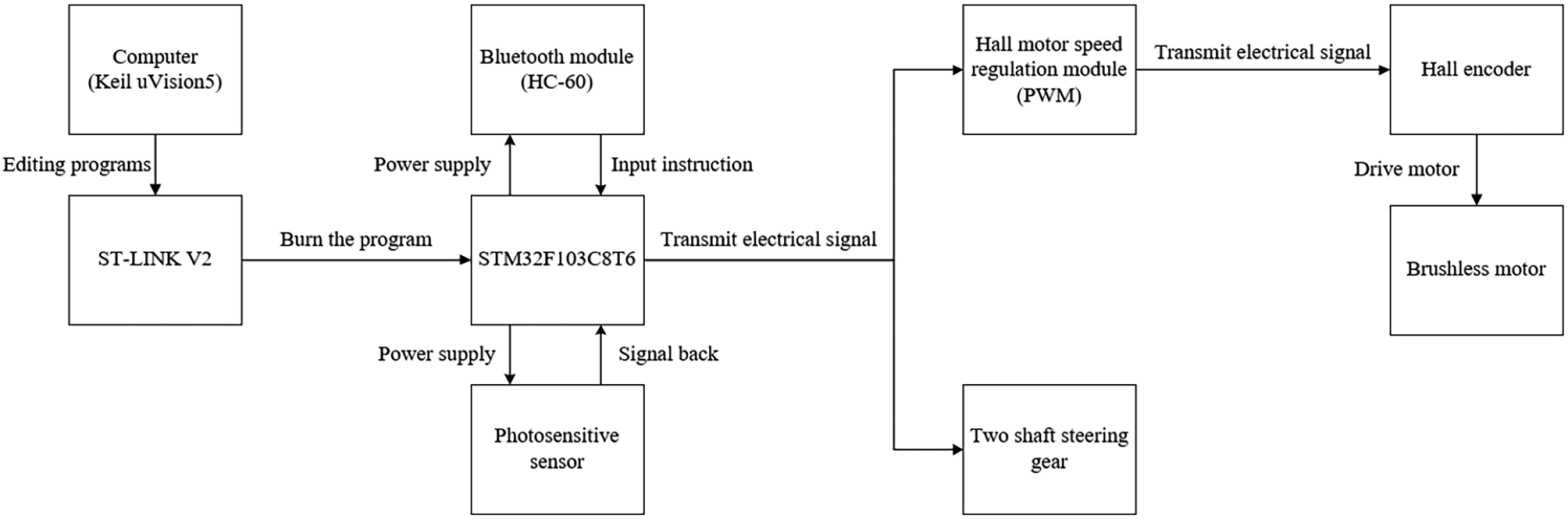
The flow diagram of the control system.

The final control effect of the machine is to regulate the speed of the brushless motor with sinusoidal waveform through the control module developed by stm32 to achieve the purpose of maintaining the steady state of flight, and to check the speed of the brushless motor through the motor speed feedback signal of the Hall encoder^19^. The control of the two-axis steering gear system is to control the steering gear through the signal transmission of the photosensitive sensor, and drive the steering gear to perform the specified state of work, and then realize the bird body jumping grab action. The main body of the control system is composed of stm32 development board, HC-60 Bluetooth module, Hall motor speed regulation module, Hall encoder and photosensitive sensor. Based on the development board, through the regulation of PWM duty ratio^20^, the equivalent analog output is obtained, and the signal output waveform is approximated to the sine curve through program adjustment. When the brushless motor obtains the equivalent analog speed signal, the Hall encoder feeds the measured motor speed signal back to the Hall motor speed regulation module, and correces the motor speed in real time through PID algorithm to achieve the purpose of correcting the speed error^21^. The photosensitive sensor acts as a switch control module in the system, which is applied to the control of leg and foot drive. When the bionic bird's foot touches the roosting target, in order to deal with the roosting failure caused by insufficient low-speed impact and deviation of landing Angle, the connected photosensitive sensor will work and transmit the signal to the development board, and then the development board will control and drive the steering gear. Perform the body leg grab. Forcing the bird's claws to be fixed on the roosting target avoids the impact damage of the body to a certain extent. At the same time, the control effect of fast response and grasping target object is achieved when the bird body lands normally. The flow diagram of the control system is shown in Fig. 13, and the physical connection of each electronic component is shown in Fig. 14.

**Figure 15:**
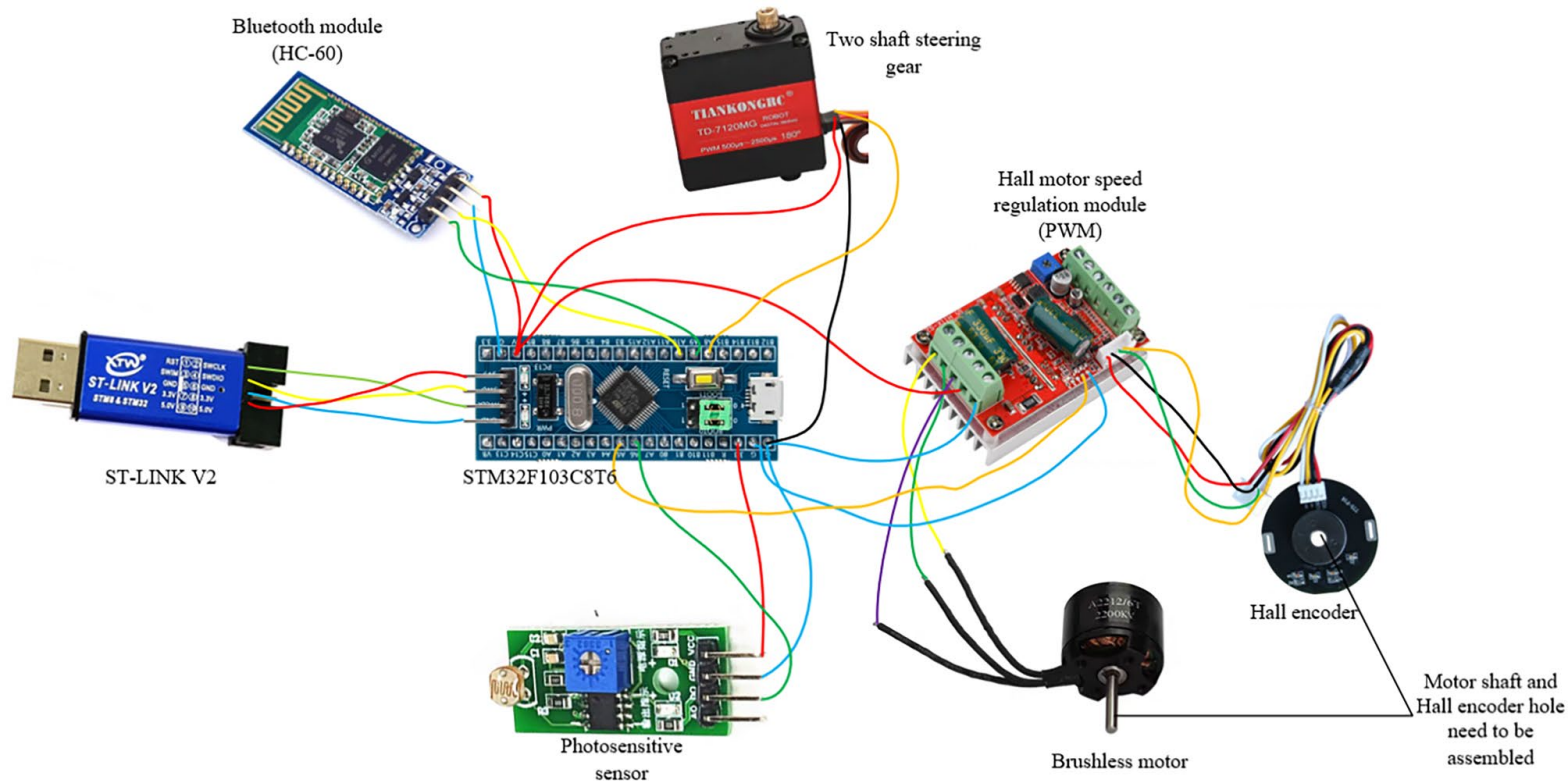
The physical connection of each electronic component.

As can be seen from Fig. 15, the main control components of the stm32 development board are the brushless motor and the two-axis steering gear. For the motor, it drives the brushless motor to speed up the wing flutter system, while for the two-axis steering gear, it drives the two-axis steering gear to rotate according to the prescribed instructions according to the feedback signal of the photosensitive sensor, and then makes the lower limb mechanism of the flapping wing to bounce and grasp with bent legs.

**Figure 16:**
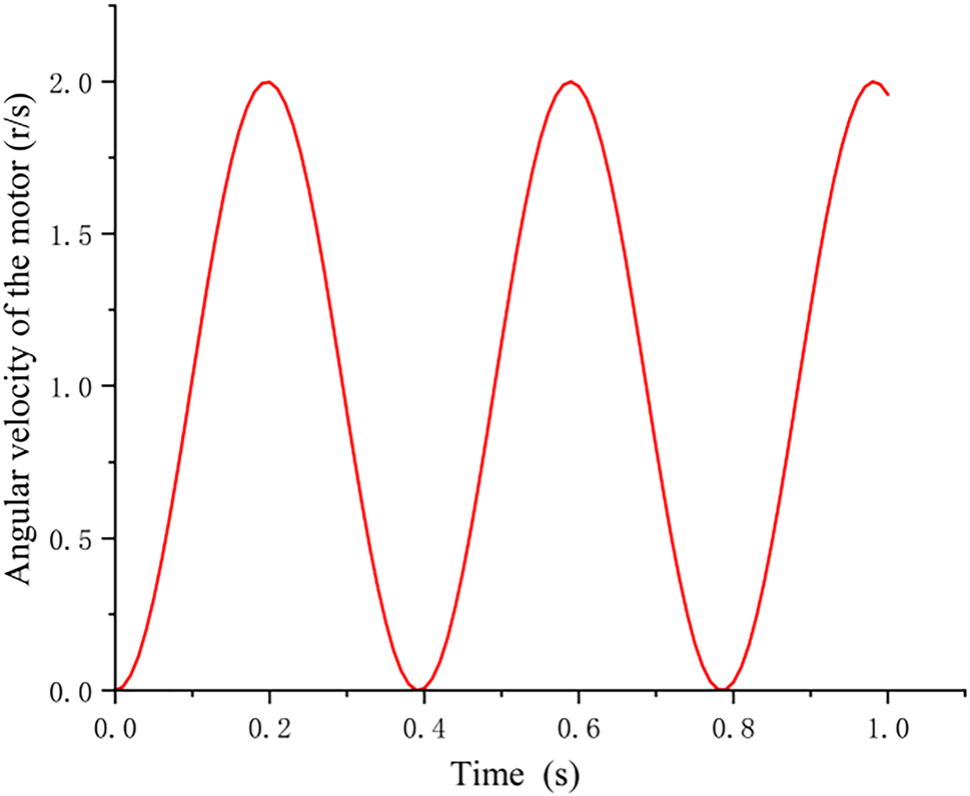
Real-time change of speed regulation waveform state.

In order to simulate sinusoidal motor speed regulation, the simulation experiment of speed regulation is carried out, and the sinusoidal law is approximated by modifying PWM duty cycle continuously. Because the up-and-down stroke of the bird's wings needs more driving force during the transition, and it wants to save electricity in the process of electric drive and improve the endurance time, sinusoidal waveform speed regulation is selected to simulate the ideal flutter action posture. Due to the influence of many uncontrollable external factors on the motor speed in the process of movement, PID algorithm is needed to adjust and control the speed through proportion, integral and differential, and finally achieve the purpose of correcting the external error. The state change of the speed regulation waveform is shown in Fig. 16.

**Figure 17:**
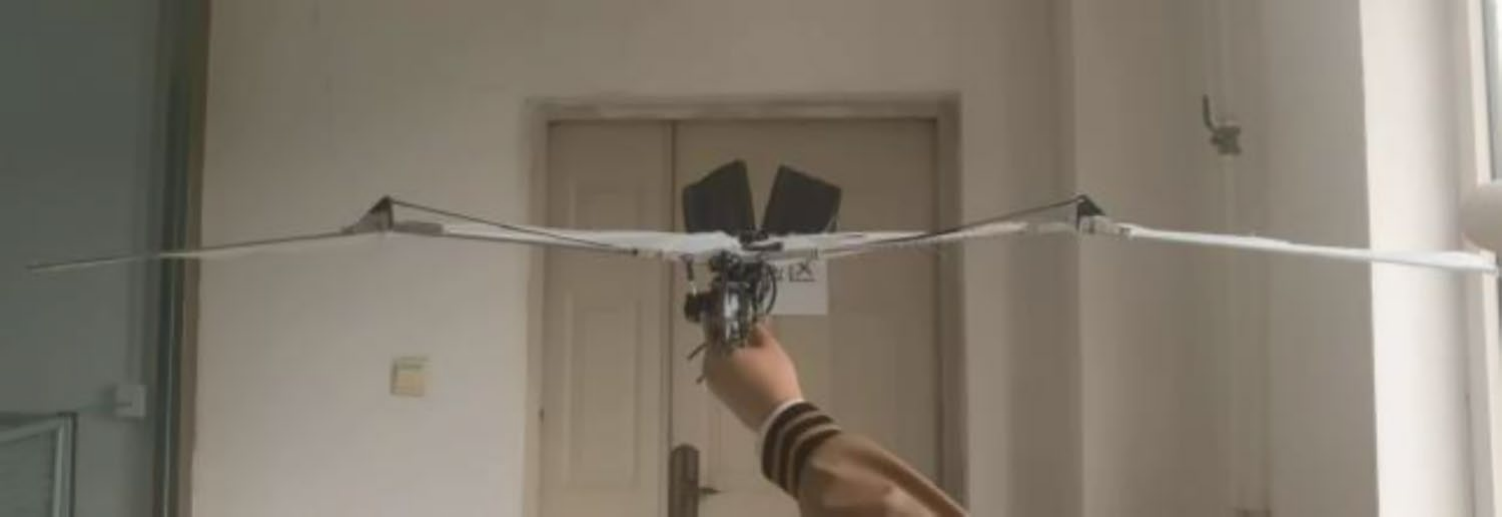
Passive deformation of wings flapping with sinusoidal speed regulation in baseline scenarios of the ornithopter.

**Figure 18:**
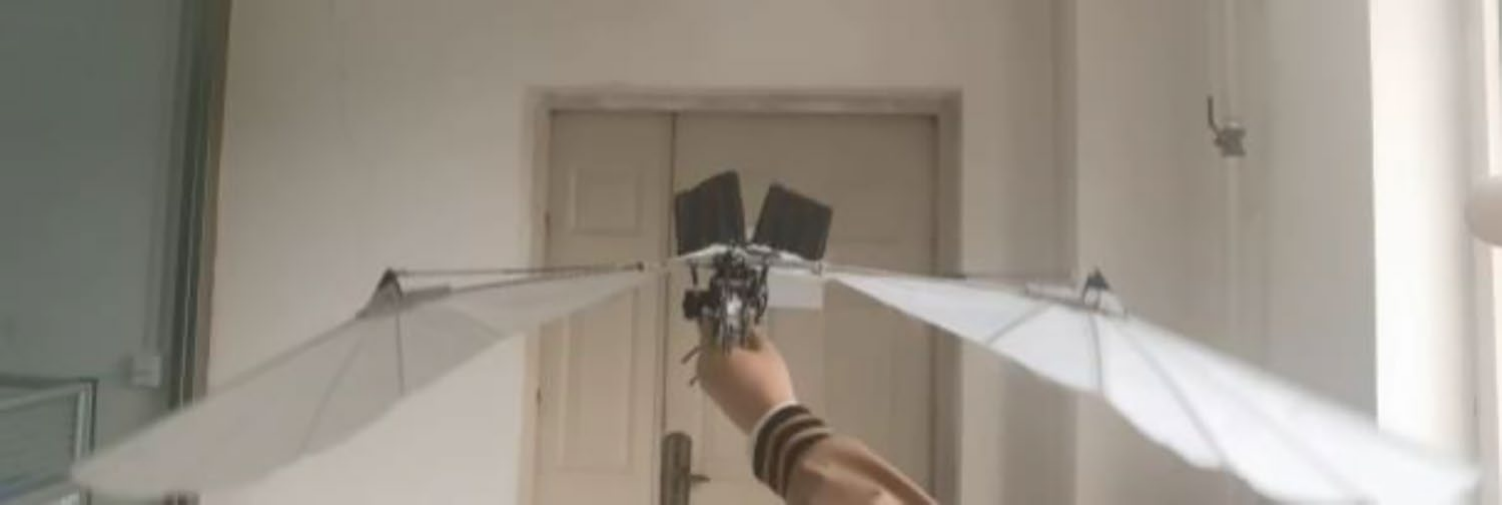
Passive deformation of wings flapping with sinusoidal speed regulation in downswing stage of the ornithopter.

**Figure 19:**
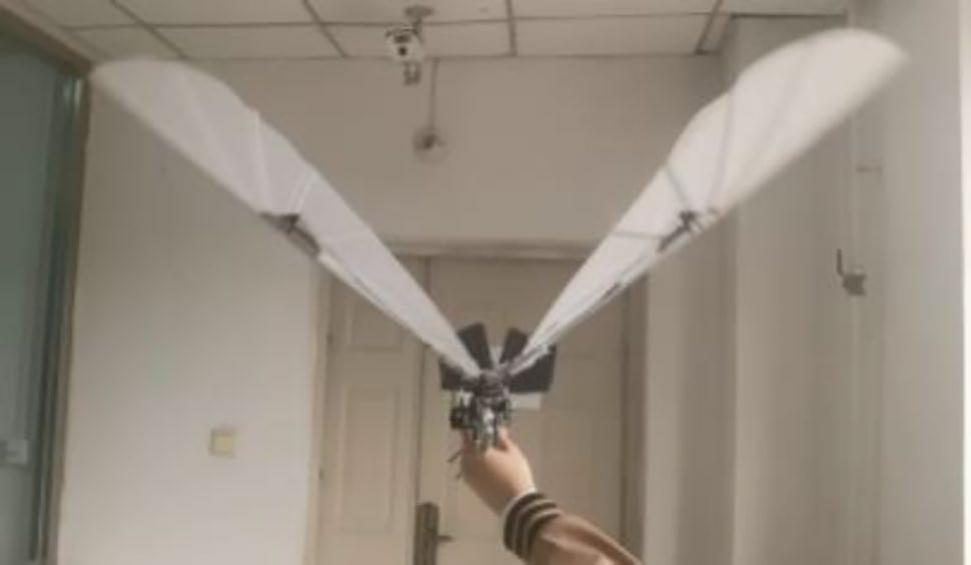
Passive deformation of wings flapping with sinusoidal speed regulation in upswing stage of the ornithopter.

After motor speed regulation, the passive deformation of wings is improved, and the push-lift ratio of bird body is improved to some extent. Compared with the flutter state without speed adjustment before, the flight attitude of the wings is more realistic and the energy utilization efficiency is higher. The deformation state of the wings compared with speed adjustment is shown in Figs. 17, 18 and 19.

**Figure 20:**
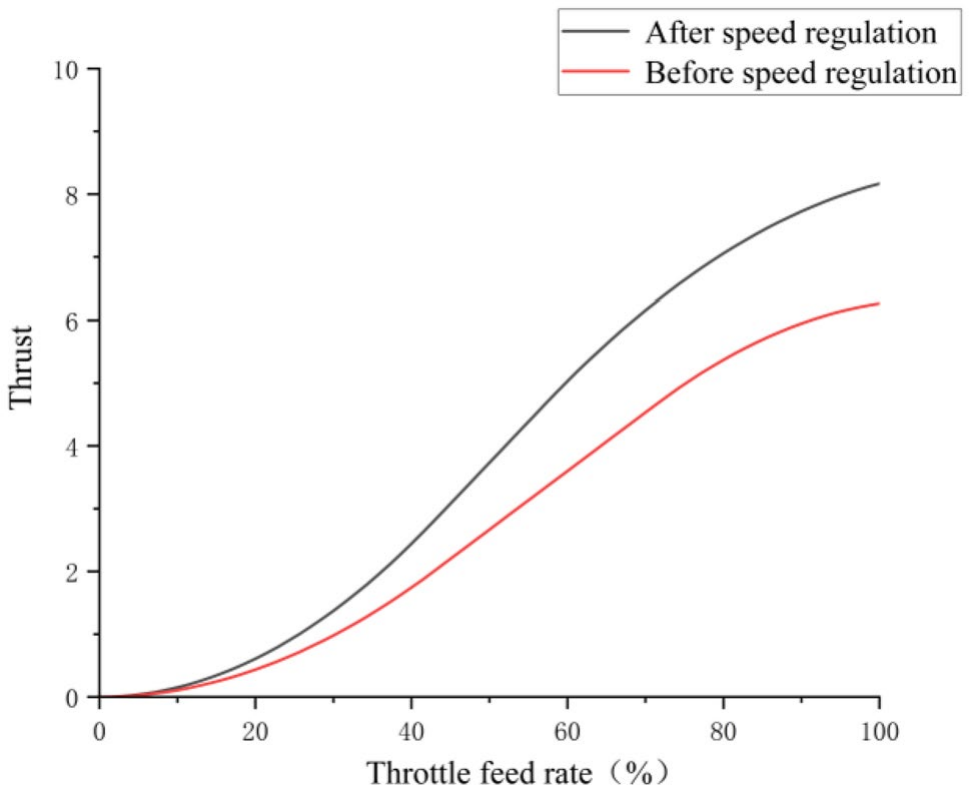
The comparison of thrust data of flapping wing before and after motor speed regulation.

The comparison of thrust data of flapping wing before and after motor speed regulation is shown in Fig. 20.

From the thrust comparison data before and after speed regulation in Fig. 20, it can be seen that the flapping wing after speed regulation can generate greater thrust under the condition of the same throttle feed, which optimizes the initial take-off condition of the flapping wing and provides the possibility for the flapping wing's bouncing take-off action.

The experiment shows that the passive deformation amplitude of wings is larger and the pusher ratio is higher after PID sine speed regulation.The horizontal initial velocity of the flapping wing body can be quickly increased, and the lift of the body can be indirectly increased, so as to complete the bouncing take-off. If the horizontal initial speed does not reach the required standard, then the flapping wing body will fail to take off due to the lack of lift, which will limit the integration of the legs and PAWS of the lower limbs of the flapping wing, so the speed regulation of the motor is essential.

## Contribution

The bionic ornithopter can be used as a reference for the lower limb structure design of bird body in the field of ornithopter wing. Its components include the design of lower limb transmission system, control system and mechanism locking system. This structure is a lightweight design based on the lack of load capacity of the flapping wing at present, which can improve the success rate of the flapping wing with load under certain conditions. In addition, the grasp function of bouncing take-off and landing in this paper is also a small step on the road to improve the bionic similarity of the flapping wing, and lays a certain foundation for the subsequent design of the bionic flapping wing lower limb structure.

A "goshawk" type bouncing flapping wing aircraft carries the lower limb structure, adopts the "splint" structure frame to build the lower limb structure loading platform, and improves the lower limb and leg characteristics of the bionic flapping wing under the premise of realizing the bouncing take-off function. In addition to the most basic copying, the incorporation of the lower limb structure improves the traditional hand-drop take-off, and can achieve fixed-point reconnaissance, reduce power loss, and extend flight time in military applications. The key technologies include: the research of the bone structure of the lower limbs of birds, the research of the impact resistance of the composition materials and the design of the transmission control system.

## Conclusion

1. The grasping reliability of the four-toe three-toe claw type is verified through the comparative grasping experiment of different claw types. The stability and fast response of the claw type are reflected from the grasping response time and the grasping force.
2. The PID algorithm of the stm32 development board is used to control the drive motor. In order to imitate the real flight condition of birds, the sine wave speed regulation algorithm is used to simulate the motor control. The experiment shows that the passive deformation of wings after PID sine speed regulation is more significant, which improves the push-lift ratio of the body and makes the flight state more stable.
3. The structure of the legs and feet of the lower limbs can achieve the expected function, and the structure design is reasonable, which can simulate the jumping and grasping action of actual birds.

## Data Availability

All data generated or analysed during this study are included in this published article.
